# IRIS: a method for reverse engineering of regulatory relations in gene networks

**DOI:** 10.1186/1471-2105-10-444

**Published:** 2009-12-23

**Authors:** Sandro Morganella, Pietro Zoppoli, Michele Ceccarelli

**Affiliations:** 1 Department of Biological and Environmental Sciences, University of Sannio, Benevento, Italy; 2 Biogem s.c.a.r.l., Ariano Irpino (Avellino), Italy

## Abstract

**Background:**

The ultimate aim of systems biology is to understand and describe how molecular components interact to manifest collective behaviour that is the sum of the single parts. Building a network of molecular interactions is the basic step in modelling a complex entity such as the cell. Even if gene-gene interactions only partially describe real networks because of post-transcriptional modifications and protein regulation, using microarray technology it is possible to combine measurements for thousands of genes into a single analysis step that provides a picture of the cell's gene expression. Several databases provide information about known molecular interactions and various methods have been developed to infer gene networks from expression data. However, network topology alone is not enough to perform simulations and predictions of how a molecular system will respond to perturbations. Rules for interactions among the single parts are needed for a complete definition of the network behaviour. Another interesting question is how to integrate information carried by the network topology, which can be derived from the literature, with large-scale experimental data.

**Results:**

Here we propose an algorithm, called inference of regulatory interaction schema (IRIS), that uses an iterative approach to map gene expression profile values (both steady-state and time-course) into discrete states and a simple probabilistic method to infer the regulatory functions of the network. These interaction rules are integrated into a factor graph model. We test IRIS on two synthetic networks to determine its accuracy and compare it to other methods. We also apply IRIS to gene expression microarray data for the *Saccharomyces cerevisiae *cell cycle and for human B-cells and compare the results to literature findings.

**Conclusions:**

IRIS is a rapid and efficient tool for the inference of regulatory relations in gene networks. A topological description of the network and a matrix of gene expression profiles are required as input to the algorithm. IRIS maps gene expression data onto discrete values and then computes regulatory functions as conditional probability tables. The suitability of the method is demonstrated for synthetic data and microarray data. The resulting network can also be embedded in a factor graph model.

## Background

Although all cells of an organism contain the same DNA, each cell only transcribes and translates a fraction of that DNA. Each cell has a particular interaction pattern that involves genes, proteins and molecules; these complex schema are known as gene regulatory networks. Full understanding of gene interactions can be used to identify methods to control the behaviour of genes directly involved in disease processes. Methods used to infer patterns of interaction among molecular components from observed data are called reverse engineering algorithms. Even if gene-gene interactions only partially describe real networks because of post-transcriptional modifications and protein regulation, using microarray technology it is possible to combine measurements for thousands of genes into a single analysis step that provides a picture of cell gene expression. Therefore, many reverse engineering algorithms rely on gene expression data [1]. These methods differ in the type of expression profiles used, so there are algorithms for time-series data [2], algorithms for steady-state data [3,4] and algorithms that work on both types of data [5]. A graphical representation of the gene regulatory network is often used, with Bayesian networks probably the most popular graphical models used in this scenario [1,5-8]. The major limitation of Bayesian networks is that they cannot represent cyclic structures. To overcome this limitation, methods based on dynamic Bayesian networks have been proposed [9-13].

However, the network topology alone is not enough to perform simulations and predictions of how a molecular system will respond to perturbations. The rules for interactions among the single parts are needed for a complete definition of the network behaviour. Another interesting question is how to integrate the information carried by the network topology, which can be derived from the literature, with large-scale experimental data. Much attention has recently been focused on the modelling [14] and inference [15] of activation rules between molecular components in the cell. Although this can be considered a simpler problem than inference of the network topology, it is important to point out the following:

• Many interaction patterns between molecular components can be obtained from the literature (using, for example, databases such as Ingenuity Pathway Analysis) and integrated in the inference algorithm.

• Owing to the limited amount of experimental data, it can be convenient to solve a simplified problem and exploit as much as possible prior knowledge about the phenomenon being investigated.

• Modelling of the interaction pattern between molecular components can be useful when performing large-scale simulations and deriving hypotheses about the behaviour of biological systems under different conditions [14].

In the present study we follow this research direction. However, instead of considering continuous expression levels, as in [15], we use a discrete representation of gene activation. Indeed, methods based on graphical models often work on discrete data obtained from real-valued gene expression profiles. The *discretisation *step is of fundamental importance for good accuracy of subsequent computational steps. Ślęzak and Wróblewski proposed a discretisation approach based on rough set theory in which quality functions are used for roughly discretised data, with inexact dependence between attribute rankings [16]. Friedman *et al. *considered two different approaches to discretise real-valued data [1]. In the first approach they discretised expression levels to several discrete states according to a fixed discretisation rule and demonstrated that this approach is sensitive to the discretisation procedure. In the second method they combined a linear regression model with the model dependence and measurements, but this approach was strongly affected by the linear dependence. Pe'er *et al. *proposed a new discretisation procedure for each gene in which gene-specific variation is used to estimate the normal distribution mixture by standard *k*-means clustering [8]. A probabilistic approach is used to identify interactions between genes, such as activation and inhibition, so this method provides a description of the gene network with the interaction features, but many of these interactions are undirected. Moreover, the discretisation step is sensitive to the choice of the number of states that a gene may attain. In this paper we propose a new discretisation approach that depends on the expression profile data (both time-course and steady-state values) for each gene, so that different genes with different expression profiles lead to different discretisation rules. Indeed, we also use this approach to reduce the effect of noise.

After discretisation, the problem faced is how to infer the rules, and not just the pattern, by which the various molecular components interact with each other. We call this problem the inference of regulatory relations given a well-specified gene regulatory network. Gat-Viks *et al. *proposed an approach to learn improved regulatory functions from high-throughput data using a discrepancy score in which discretisation is carried out as a preprocessing step [17], but the discretisation rules must be determined and tuned rather arbitrarily and each variable is discretised using the same rule. Gat-Viks *et al. *also proposed a more flexible approach to learn the regulatory functions in a gene network [18], which is represented as a factor graph [19] to model cyclic structures. In this approach discretisation is carried out according to an expectation maximisation (EM) algorithm that, combined with the factor graph model, provides a very flexible discretisation scheme. However, in practice this flexibility can lead to over fitting and may decrease learnability, so the authors suggested to use the same or a few discretisation schemes for all the variables. Chuang *et al. *proposed an approach to infer gene relations from time-course expression profiles in which first and second derivatives are used to detect time-lagged correlated gene pairs [20]. The basic assumption is that pairs of correlated genes exhibit either a complementary pattern (that represents a repressor relation) or a similar pattern (that represents an activator relation). In our approach we propose a simple regulatory function inference that is particularly fast and yields rather good accuracy, even if the network is cyclic. In this step we use an observation similar to that of Chuang *et al. *[20] for expression profile patterns, but here we use discrete data. Finally, we merge the inferred regulatory functions into a factor graph representation as reported by Gat-Viks *et al. *[18].

## Implementation

### Biological Model

We first define a simple model for biological networks [17]. A biological network can be modelled through a direct graph *G*(*V*, *E*), where each node *v *∈ *V *represents a gene that can be in a discrete state *D *= {0,1}, representing an inactive and an active state, respectively. If a gene *v *∈ *V *has at least one parent then it is called a *regulated gene *and we define as *R*_*v *_the set of parents (*regulators*) of *v*. If a gene *v *∈ *V *has no parents, then it is a *stimulator *and we define as *V*_*s *_the set all stimulators of the network. In addition, we represent the expression data using an *n *× *m *matrix *M*, where *n *is the number of genes in the network and *m *is the number of experiments performed or samples. For each 1 ≤ *i *≤ *n *and 1 ≤ *j *≤ *m *the value of *M *[*i*, *j*] is the expression level of gene *i *in sample *j*.

### IRIS Algorithm

In this section we describe our approach to infer the regulatory relations in gene networks from high-throughput data. IRIS needs an input network topology *N*, an expression profile data matrix *M*. The method consists of two main steps: *(i) *Dicretisation and *(ii) *Regulation Functions Learning. The details are reported in the following subsections.

#### Discretisation

This steps is aimed at computing a binary matrix from the observed gene activation levels. The discretisation step uses a matrix of local variation of the gene expression, defined as  = *M*_*s*_[*i*, *j*] - *M*_*s*_[*i*, *j *- 1], *j *= 2,..., *m*, Let *S *be the matrix of discrete states, i.e. *S *[*i*, *j*] contains the discrete state relative to the value *M*_*s *_[*i*, *j*]. The discretisation procedure is iterative. It first tries to fix the lower values to zero and the upper values to one on the basis of two thresholds  and , they are computed in order that the interval [, ], for each row *i*, contains a given percentage, *α*, of the second and third quartiles of the data. The other values are then fixed on the basis of their nearby values.

We extensively tested various values of *α *for various datasets, and the results showed that it can be arbitrarily chosen in the range 5%-35% without significantly affecting the results. In all the experiments reported below we choose *α *as the minimum of this range.

We compute the first discretisation step using the rule:(1)

with 2 ≤ *j *≤ *m*. And for *j *= 1 we use:(2)

**Figure 1:**
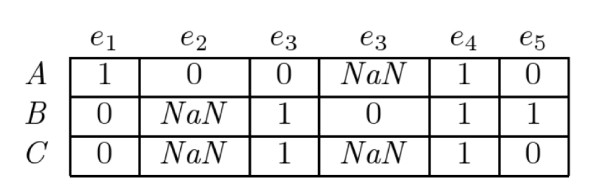
**Example of the discrete state matrix *S***.

**Figure 2:**
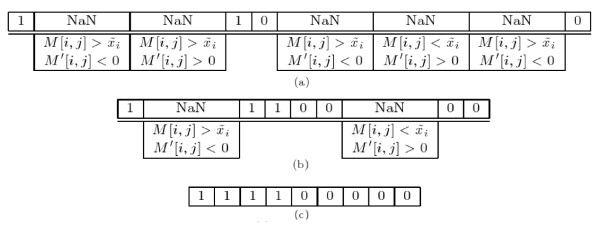
**Recovery step example**. (a) Data obtained by the discretisation rule defined in (1). (b) Data obtained after one iteration of the rules defined in (3) and (4). (c) Final data for which all uncertain values were recovered.

Figure 1 shows an example of matrix *S *for three genes *A*, *B *and *C*. The values of *S *computed as above are used to compute neighbouring values. In particular, the uncertain values (*NaN*) are recovered as follows:(3)

with 2 ≤ *j *<*m *- 1 and where  is the median value for the expression of gene *i*. To apply these rules we use an iterative approach that runs until either all values are assigned to a valid active/inactive state or no recovery action is performed in the last iteration. Figure 2 shows an example of recovery computation.

#### Regulation Functions Learning

In this step we use the matrix *S *to compute the PTs and TTs. To infer the PTs we use relative frequencies. Consider a gene *v *and the set of its regulators *R*_*v*_. Then the matrix *S *contains several state assignments for the genes in *R*_*v *_and *v *itself. Let Γ_*v *_be the set of all possible state assignments of the variables in *R*_*v*_.

Then we have:(5)

where |{*r*_*v*_, *v *= *s*}| are the occurrence numbers of state assignment {*r*_*v *_∪ *v *= *s*} in *S*. Let

PT({*v *= *s*}|{*r*_*v*_}) be the conditional probability that gene *v *is in state *s *given the state assignment *r*_*v *_∈ Γ_*v*_. Then we compute the conditional probabilities for *v *as follows:(6)

Using (6) we compute the TT for each gene as:(7)

∀ *r*_*v *_∈ Γ_*v *_and where TT({*r*_*v*_}) represents the state response of regulated gene *v *to the state assignment *r*_*v *_of its regulators. Note that if

we cannot distinguish between the active and inactive state, so we have an undefined response of regulated gene *v *to the state assignment {*r*_*v*_}. This situation is indicated as -1 in the TTs.

### Integration with Factor Graph

A factor graph is a class of probabilistic models that were originally applied to coding/decoding problems. Using a factor graph we can model complex domain knowledge in which feedback loops play a fundamental role. One of the important advantages of factor graphs is their combination with the sum-product algorithm [19], which is a message-passing algorithm for efficiently computing marginal distributions, even in the presence of cycles.

**Figure 3:**
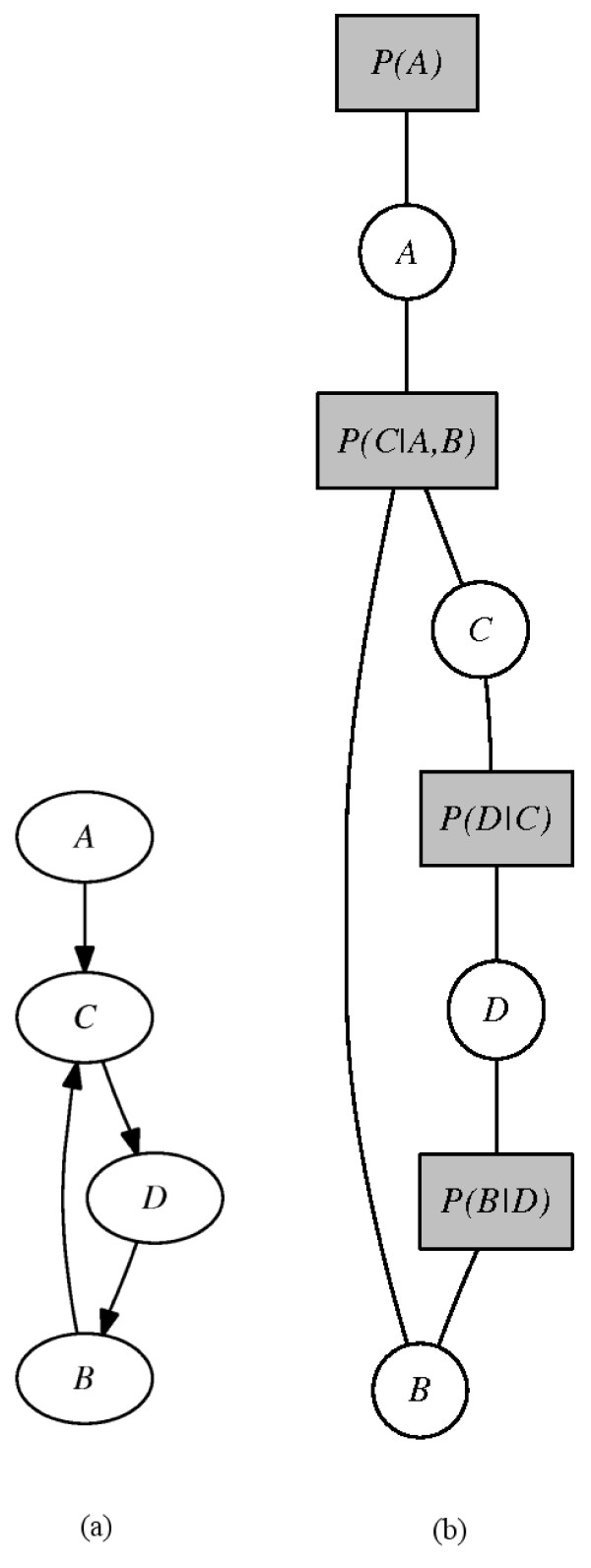
**Modelling the gene regulatory network as a factor graph**. (a) Gene regulatory network modelled as a direct graph. (b) Equivalent factor graph representation of the network in (a).

In our approach the factor graph model is used to combine structural information (network topology) with the inferred regulatory functions. A factor graph contains two types of nodes: *factor *and *variable *nodes. We have a variable node for each gene and a factor node linking two variable nodes if and only if a relationship between these two nodes exists. Consider the gene regulatory network depicted in Figure 3(a) containing four genes: *A *and *B *are regulators of *C*, *C *is a regulator of *D*, and *D *is regulator of *B*. Note that gene *A *is a stimulator of the network. Since there are three regulated genes, we have three PTs representing *P*(*C*|*A*, *B*), *P*(*D*|*C*) and *P*(*B*|*D*). To translate this gene network into an equivalent factor graph network, we perform the following steps:

• Each gene node becomes a variable node of the factor graph;

• For each regulatory function a factor node must be inserted to link the genes involved;

• Each stimulator must be linked to a specific factor node.

Applying these rules, we obtain the factor graph in Figure 3(b). This model can answer questions such as: "What is the probability that gene *C *is active given that genes *A *and *B *are inactive?" and "What is the likelihood that genes *B *and *A *are inactive given that gene *C *is active?". For this purpose, we set the state of the observed genes and use the sum-product algorithm to compute the posterior distribution of hidden genes. Here we follow the propagation of belief in directed graphs for Forney-style factor graphs [21]. If a stimulator is not fixed, then its factor node will be set with a uniform distribution.

Some considerations about the presence of cycles are useful, indeed this is one of the most important problems in the field of Probabilistic Graphical Models (PGM). The inference in PGM consists in the computation of marginal probabilities of complex probability distributions defined over many variables. Many exact and approximate algorithms have been proposed for this task [22] from Monte Carlo methods to variational methods, mean field methods and belief propagation (BP). The sum-product algorithm, adopted in this paper, is a special case of belief propagation. It is well-known that belief propagation yields exact results if the graphical model does not contain cycles. If the graphical model contains loops, the sum-product algorithm can still yield accurate results using little computational effort [21]. However, if the influence of loops is large, the approximate marginals calculated by BP can have large errors and the quality of the BP results may not be satisfactory. Many recent research efforts in statistical machine learning are devoted to the development of efficient approximate inference algorithms for cyclic graphical models (see for example [23]). For the purposes of this paper we could have to deal with cycles in the case of inference, when we use PTs, and computation of steady states, when using TTs. We adopt the belief propagation algorithm for the former and the algorithm of Gat-Viks *et al. *[17] for the latter. In any case the IRIS method, proposed here, is by no way influenced by the presence of cycles. In particular IRIS takes as input the description of the network and the expression profiles giving in output a map of the regulatory relations between sets of regulators and regulated genes, this means that all the information used by IRIS are based on "local" relationships between a gene and the set of its regulators. The influence of cycles appears in the successive phases for the use of these relationships in inference tasks. However, the inference in cyclic PGMs is still a very important research question in the field of statistical machine learning and its solution is, of course, outside the scopes of this paper.

## Results

In this section we report IRIS results for both synthetic networks and microarray expression profiles. IRIS needs a well-defined gene network as input. We say that a gene network is well defined if each of its interactions allows us to distinguish between *regulator *genes and *regulated *genes. Given a well-defined network, we have genes with zero regulators (called *stimulators *representing environmental conditions), genes with one regulator, genes with two regulators, and so on. If a gene has at least one regulator then it has a regulatory function that describes its response to a particular stimulus by its regulator(s). In our approach we suppose, without loss of generality, that a gene can be in one of two states: inactive and active, represented as 0 and 1, respectively. This assumption is commonly used in the literature to distinguish the response of a gene to a given experimental condition.

Given a well-defined gene regulatory network, IRIS computes the regulatory functions, providing two different descriptions: a description in which each interaction is described as a conditional probability table, which we refer to as a *potential table *(PT), and a description where each regulatory relation is a truth table, which, by analogy to neural logic networks, we refer as a *truth table *(TT). These two different descriptions allow different analyses. Using the PTs we can execute an inference step to compute the *a posteriori *probability of hidden genes given observed genes, so that, for example, it is possible to understand how to control a gene using particular environmental conditions. Using the TTs we can compute the steady states of a gene regulatory network. In this scenario, we deal with the problem of the cyclic structure of gene networks, so we use an approach based on the factor graph model [19] as an inference engine and the idea of feedback sets [17] to compute the steady states of the networks.

### Results for Synthetic Networks

**Figure 4:**
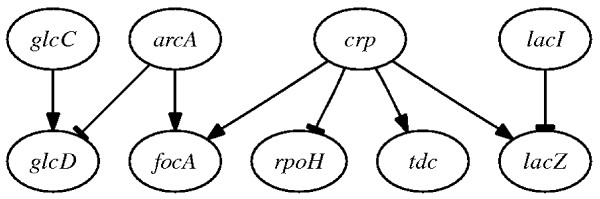
**Synthetic network for *E. coli***.

**Figure 5:**
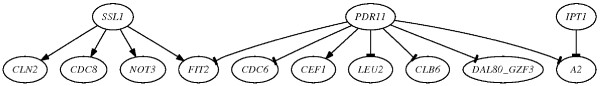
**Synthetic network for *S. cerevisiae***.

The synthetic networks used to test IRIS were generated using SynTReN [24], it creates synthetic transcriptional regulatory networks and produces simulated gene expression data that approximate experimental data. Network topologies are generated by selecting sub-networks from previously described regulatory networks. Several parameters can be used to adjust the complexity of the data set generated. All gene expression values are normalized between 0 (no transcription) and 1 (maximum level of transcription). In addition, the data generated can be altered by a specified level of biological and experimental noise. Using SynTReN, we generated two synthetic networks representing a sub-pathway of the *E. coli *regulatory-network (Figure 4) and a sub-pathway of the *S. cerevisiae *regulatory network (Figure 5). For both synthetic networks we generated a data set 150 samples for each biological noise level in the set {0.10,0.15,0.20,0.25,0.30,0.35,0.40, 0.45,0.50}, using experimental noise levels of 0.18 and 0.25 for *E. coli *and *S. cerevisiae*, respectively.

Most of the rules governing the activation of genes in these networks have already been investigated in several studies. In particular, for the *E. coli *network we use conclusions from references [25-28] to obtain the regulatory relations for *glcD, focA *and *lacZ*. For *S. cerevisiae *we use the results of Wilcox *et al. *[29] to obtain the regulatory relation for *FIT2*. Since all the regulators of *A2 *have an inhibitory function, the gene will be in an active state if and only if both regulators are inactive. For more details on true descriptions, see Additional file 1: regulation_true_descriptions.pdf.

**Figure 6:**
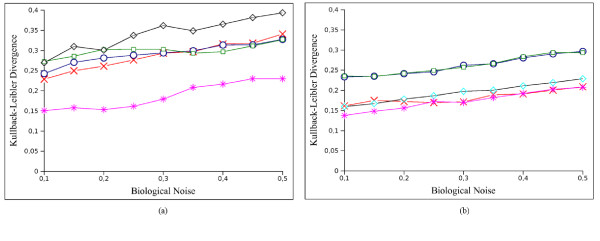
**Comparison Kullback-Leibler divergence for (a) *E. coli *and (b) *S. cerevisiae *synthetic networks**. The *x*-axis shows the biological noise levels used to generate the data sets. The *y*-axis represents the *D*_*KL *_values obtained as the mean for all tables of a network for the corresponding biological noise level. The Figure reports the *D*_*KL *_values of EM-MAP obtained using different discretisation approaches: IRIS (crosses), equal frequency (diamonds), global width (squares) and equal width (circles) and the *D*_*KL *_values obtained using IRIS algorithm both in discretisation step and in regulation function learning process (asterisks).

Using IRIS we inferred the regulatory functions for both synthetic gene networks. To evaluate the accuracy of the PTs computed by IRIS, we used the Kullback-Leibler divergence (*D*_*KL*_) [30], which is a measure of the difference between two probability distributions *P *and *Q*, with *D*_*KL*_(*P*||*Q*) = 0 if and only if *P *= *Q*. Figure 6 shows mean *D*_*KL*_(*P*_*true*_||*P*_*IRIS*_) values as a function of the noise level (asterisks). The results in terms of correct TT entries inferred by the algorithm are reported in Table 1 for a maximum biological 0.5. The size of each regulation table, which depends on the number of regulator genes, is also reported. If |*R*_*i*_| is the number of regulators of the *i*th gene, then the regulation TT will have a size of , assuming that a gene can be in two states. The results show that all but one activation rule were correctly inferred by IRIS.

**Table 1:** Percentage of correct entries in the inferred truth tables for synthetic networks for *E. coli *and *S. cerevisiae*.

E. Coli					
			**True Table vs IRIS Inferred TT**		
			
**Regulated Gene**	**Regulator Genes**	**TT Size**	**Correct**	**Incorrect**	**Undefined**

*glcD*	*glcC arcA*	4	4	0	0

*focA*	*arcA crp*	4	4	0	0

*rpoH*	*crp*	2	2	0	0

*tdc*	*crp*	2	2	0	0

*lacZ*	*crp lacI*	4	4	0	0

Total		16	16	0	0

Percentage			100%	0%	0%

**S. cerevisiae**					

			True Table vs IRIS Inferred TT		
			
Regulated Gene	Regulator Genes	TT Size	Correct	Incorrect	Undefined

*CLN2*	*SSL1*	2	2	0	0

*CDC28*	*SSL1*	2	2	0	0

*NOT3*	*SSL1*	2	2	0	0

*FIT2*	*SSL1 PDR11*	4	4	0	0

*CDC6*	*PDR11*	2	2	0	0

*CEF1*	*PDR11*	2	1	1	0

*LEU2*	*PDR11*	2	2	0	0

*CLB6*	*PDR11*	2	2	0	0

*DAL80_GZF3*	*PDR11*	2	2	0	0

*A2*	*PDR11 IPT1*	4	4	0	0

Total		24	23	1	0

Percentage			95.83%	4.17%	0%

Truth tables (TTs) were computed using the data set with the maximum biological noise level (0.50). "TT Size" reports the number of possible state assignments for the regulator genes. Note that the value for the i-th regulated gene is , where |R*i*| is the number of regulators and each gene can be in two states. We distinguish the number of correct/incorrect/undefined inferred states for each regulatory relation and compute these as percentages of the total number of states.

Both networks generated by SynTReN have an acyclic structure, so we can use the EM-MAP algorithm [31] to compare the performance of IRIS. We used an EM-MAP algorithm implementation of the Bayes net toolbox (BNT) [32] to validate the IRIS discretisation scheme. Figure 6 shows results for different discretisation methods such as equal frequency (diamonds), global width (squares) and equal width (circles). For these methods we used discretisation into two bins. The EM-MAP results for IRIS discretisation are reported in Figure 6 (crosses). The mean values over all levels of biological noise are:

1. *D*_*KL *_(*P*_*true*_||*P*_*IRIS*_) = 0.1872 and *D*_*KL *_(*P*_*true*_||*P*_*EM*-*MAP*_) = 0.2865 for *E. coll*

2. *D*_*KL *_(*P*_*true*_||*P*_*IRIS*_) = 0.1743 and *D*_*KL *_(*P*_*true*_||*P*_*EM*-*MAP*_) = 0.1821 for *S. cerevisiae*.

Table 2 lists the execution times (taken using a Linux PC with Intel Core Duo CPU at 1.8 Ghz) for IRIS and EM-MAP. We report the running times not for an absolute evaluation, but for a relative comparison, indeed the proposed method yields similar or slightly better results than EM-MAP and requires less computational resources. Actually the running time of IRIS depends linearly on the number of genes, and the number of samples, however it depends exponentially from the maximum in-degree of the node network.

**Table 2:** Execution time for IRIS and EM-MAP.

	*E. coli*			*S. cerevisiae*		
	
*Execution Time*	IRIS	EM-MAP		IRIS	EM-MAP	
**Biological Noise**	**Time**	**Time**	**Iter**	**Time**	**Time**	**Iter**

0.10	0.929 s	9.447 s	6	1.833 s	14.557 s	5

0.15	0.897 s	9.435 s	6	1.892 s	14.399 s	5

0.20	0.910 s	8.770 s	5	1.853 s	14.510 s	5

0.25	0.905 s	8.642 s	5	1.874 s	14.381 s	5

0.30	0.968 s	8.787 s	5	1.876 s	17.446 s	6

0.35	0.965 s	8.762 s	5	1.790 s	17.432 s	6

0.40	1.041 s	8.880 s	5	1.825 s	17.469 s	6

0.45	0.953 s	8.674 s	5	1.839 s	14.600 s	5

0.50	1.005 s	8.741 s	5	1.860 s	14.735 s	5

Each value was obtained as the mean for 10 runs. For EM-MAP we also report the number of iterations to reach convergence.

Since the IRIS discretisation scheme can be influenced by the order of the values for expression of each gene in the data set, it is interesting to investigate how the performance changes on randomly changing the order of the data. This is useful for a steady-state data set, for which the order of the expression profile has no biological meaning. Actually the difference between the expression levels at successive points of the profile has been often used to characterise the behaviour of a gene in different conditions, with particular reference to time course experiments (see for example [20]), as it can be used as an indicator of increase/decrease of expression profiles correlated in time. IRIS makes use of this difference in the discretisation procedure to fix the values which are not significantly low or high with respect to the mean expression level in the profile. Actually we found useful to compare differences in the expression levels over different conditions even for non time course data. The intuitive reason for the use of the information carried by the difference of expression levels is based of the fact that in this way we can observe if two or more genes have a common or an opposite pattern, which are indicator of activation and inhibition respectively. For example, suppose that we have a network where the gene *A *is a regulator of another gene *B *and let *E*_1 _and *E*_2 _be two different experimental conditions of a steady state dataset. Suppose also that *a*_1 _(*b*_1_) and *a*_2_(*b*_2_) are the expression levels of *A*(*B*) under *E*_1 _and *E*_2 _respectively. Now we can distinguish two different situations:

a) *a*_1 _<*a*_2 _and *b*_1 _<*b*_2 _(or *a*_1 _>*a*2 and *b*_1 _>*b*_2_): here we can state that in the experiment *E*_2 _both genes have an expression level greater (lower) then in *E*_1_, in other words, the two genes have a similar behaviour.

b) *a*_1 _>*a*_2 _and *b*_1 _<*b*_2 _(or *a*_1 _<*a*_2 _and *b*_1 _>*b*_2_): here we can state that in *E*_1 _the gene *A *has an expression level greater (lower) then in *E*_2_, whereas, the gene *B *has an expression level in *E*_1 _lower (greater) than in *E*_2_, in other words, the two genes have an opposite behaviour which can be observed form an opposite sign of the expression derivative for the genes in the experimental condition *E*_2_.

**Figure 7:**
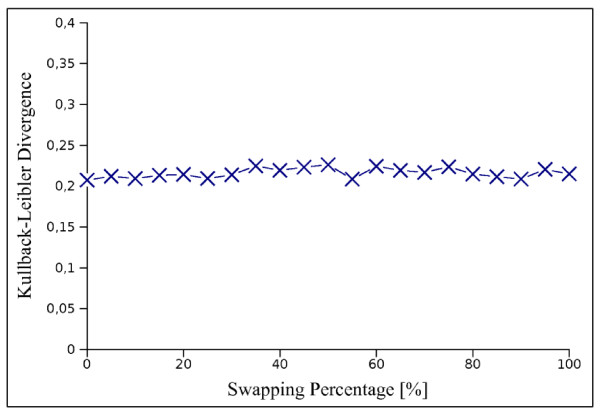
**Kullback-Leibler divergence for IRIS using randomised data sets**. The *S. cerevisiae *synthetic network and the data set with biological noise of 0.5 were used in this test. The *x*-axis represents the percentage of columns swapped randomly. The *D*_*KL *_values reported are the means for 100 runs.

If two genes show the behaviour of the case a) than we can suppose that these two genes have a relationship of activation, whereas, in the second case they have a relationship of inhibition. The fact that for steady state data we have uncorrelated values, suggests to choose any set of points to extract second order information. In order to maintain, a coherence with the case of time course data, without loss of generality, we use the previous point in the expression profile matrix. In order to demonstrate that the choice of the previous point does not significantly affect the results, we evaluated the results in terms of Kullback-Leibler divergence with the known solution on a steady state dataset performing random permutations on the expression profile of each gene. These results reported in Figure 7 show that for different permutations the Kullback-Leibler divergence is nearly constant, in other words, the choice of the previous point has the same impact on the results that could have the choice of any other point for the computation of the difference. Finally, the use of multiple points instead of just the previous one did not produce significant improvements, at the expenses of an increased computational time.

### Results for Microarray Expression Profiles

We also applied the IRIS algorithm to two real data sets comprising microarray expression profiles for the yeast mitotic cell cycle and human B-cells.

#### Yeast Mitotic Cell Cycle

**Figure 8:**
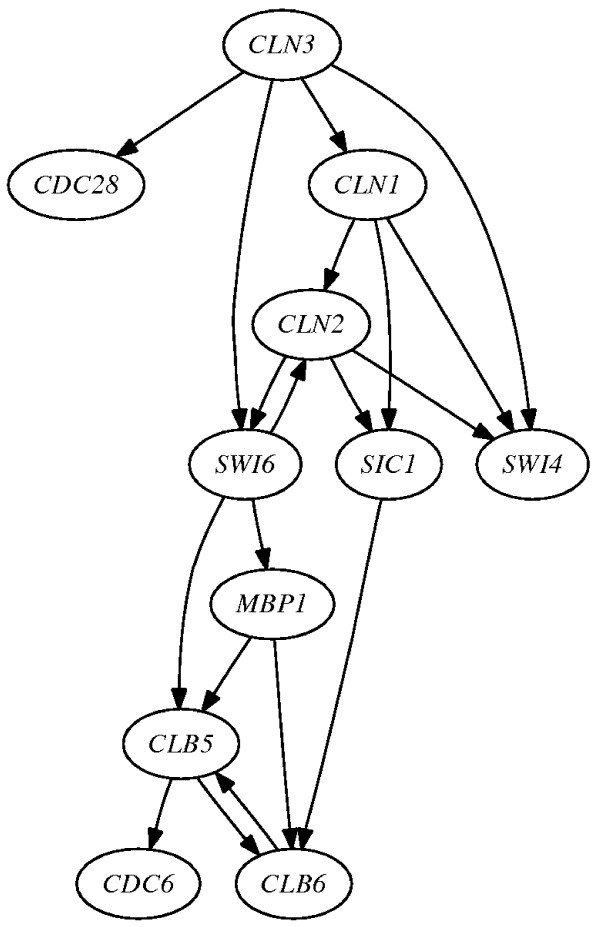
**Network for the *S. cerevisiae *cell cycle**.

Figure 8 shows the network topology for the yeast mitotic cell cycle extracted from the study by Noman and Iba [33], where we consider only known interactions reported in the literature. In this network the transcriptional factors *SWI4, SWI6 *and *MBP1 *are directly linked to the cyclins *CLN1, CLN2, CLN3, CLB5 *and *CLB6*, which bind to the cyclin-dependent kinase protein *CDC28*, whereas *SIC1 *is an inhibitor of the cyclin *CDC28 *complex. Using the literature [34,35] we obtain a description of the regulatory relations for this network (for more details see Additional file 1: regulation_true_descriptions.pdf). These relationships can be considered the truth tables for this pathway. To infer the regulatory functions we use the microarray data from Spellman *et al. *[36].

Table 3 compares literature truth tables with those inferred by IRIS. IRIS properly computes the TTs for the genes *CDC28, MBP1, CLN1, CDC6 *and *CLN2*, with an overall accuracy of 75%, an error rate of 14% and 11% undefined activations. One of the main advantages of the PT representation of the regulatory functions is the possibility of performing Bayesian inference with a belief propagation algorithm to compute the marginal distributions of *hidden genes *given *observed genes *as reported in Table 4. It is interesting that the marginal distributions in the final column of the table reflect the biological findings reported in the literature shown in the first column of the table.

**Table 3:** Percentage of correct entries in inferred truth tables for the *S. cerevisiae *mitotic cell-cycle network.

			True Table vs IRIS Inferred TT		
			
Regulated Gene	Regulator Genes	TT Size	Correct	Incorrect	Undefined
*CDC28*	*CLN3*	2	2	0	0

*MBP1*	*SWI6*	2	2	0	0

*CLN1*	*CLN3*	2	1	0	1

*CDC6*	*CLB5*	2	2	0	0

*CLN2*	*CLN1 SWI6*	4	4	0	0

*CLB 5*	*SWI6 MBP1 CLB6*	8	7	0	1

*SWI6*	*CLN2 CLN3*	4	2	2	0

*CLB6*	*SIC1 MBP1 CLB5*	8	6	1	1

*SWI4*	*CLN1 CLN2 CLN3*	8	6	1	1

*SIC1*	*CLN1 CLN2*	4	1	2	1

Total		44	33	6	5

Percentage			75%	13.64%	11.36%

This table follows the same schema as for Table 1.

**Table 4:** Inference results. Column "Biological Findings" lists a short description of the features of interest and references.

Biological Findings	Observed Genes	Hidden Genes	Inference Results
Strong relationship between cyclins *CLB5 *and *CLB6 *[40] and between *CLN1 *and *CLN2 *[41]	*CLB5*	*CLB6*	*P*(*CLB*6 = 0|*CLB*5 = 0) = 0.9851 *P*(*CLB*6 = 1|*CLB*5 = 1) = 1.0000
	
	*CLB6*	*CLB5*	*P*(*CLB*5 = 0|*CLB*6 = 0) = 1.0000 *P*(*CLB*5 = 0|*CLB*6 = 0) = 0.8079
	
	*CLN1*	*CLN2*	*P*(*CLN*2 = 0|*CLN*1 = 0) = 0.95028 *P*(*CLN*2 = 1|*CLN*1 = 1) = 0.8449
	
	*CLN2*	*CLN1*	*P*(*CLN*1 = 0|*CLN*2 = 0) = 0.6111 *P*(*CLN*1 = 1|*CLN*2 = 1) = 0.6111

Inhibitory activity of *SIC1 *on cyclins *CLB5 *and *CLB6 *[42]	*SIC1*	*CLB5*	*P*(*CLB*5 = 0|*SIC*1 = 1) = 0.7367
		*CLB6*	*P*(*CLB*6 = 0|*SIC*1 = 1) = 0.7367

Inactivation of *MBP1 *and *SWI6 *causes *CLB5 *and *CLB6 *levels to fall [35]	*MBP1*	*CLB5*	*P*(*CLB*5 = 0|*MBP*1 = 0, *SWI*6 = 0) = 0.7742
	*SWI6*	*CLB6*	*P*(*CLB*6 = 0|*MBP*1 = 0, *SWI*6 = 0) = 0.8483

While *CLN1 *and *CLN2 *are active, *SIC1 *is degraded [43]	*CLN1*	*SIC1*	*P*(*SIC*1 = 0|*CLN*1 = 1, *CLN*2 = 1) = 0.6476
	*CLN2*		

Inactivation activity of *MBP1 *and *SWI6 *on *CLN1 *and *CLN2 *[44]	*MBP1* *SWI6*	*CLN1*	*P*(*CLN*1 = 0|*MBP*1 = 1, *SWI*6 = 1) = 0.6111
		*CLN2*	*P*(*CLN*2 = 0|*MBP*2 = 1, *SWI*6 = 1) = 0.6148

To infer the conditional probability reported in the "Inference Results" column, the sum-product algorithm was used, and the state of the "Observed Genes" is fixed to derive the potential behaviour of "Hidden Genes".

Finally, the inferred TTs can be used to compute the steady states of the network using an appropriate algorithm [17]. In this case three steady states are identified (Table 5). Among them the first and the third one have been described in literature [35].

**Table 5:** Steady states for the yeast mitotic cell-cycle network obtained using IRIS

*CLN3*	*MBP1*	*SWI6*	*SWI4*	*CLN1*	*CLN2*	*CLB5*	*CLB6*	*SIC1*
0	0	1	0	0	0	0	0	0

0	0	1	0	0	0	1	1	0

1	0	0	1	1	1	0	0	0

#### Human B-Cells

Recent studies have demonstrated that the organisation of a gene regulatory network often follows a scale-free nature [37]. A scale-free network is characterised by an inverse relationship between the number of nodes and their connectivity. Another feature of gene networks is the presence of highly connected genes (called *hubs*). These networks typically contain short feedback loops. To test the suitability of IRIS for scale-free gene regulatory networks, we inferred the regulatory relations from human B-cell data, for which we considered the *MYC *gene as a major hub. *MYC *codes for a protein that binds to the DNA sequence of other genes. When *MYC *is mutated or over expressed, the protein does not bind correctly and often causes cancer. Both the gene expression profiles and network topology were extracted from the results of Basso *et al. *[3]. The network topology represents the *MYC *gene and 55 genes directly connected to it. To infer the regulatory rules of this network, we used a subset of 100 expression profiles (in [3] 336 samples are used). Here we use the *MYC *target gene database (MYC-DB) [38].

**Figure 9:**
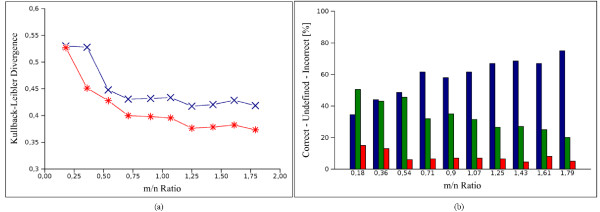
**Results on MYC-subnetwork for different *m*/*n *ratio values**. The ratio *m*/*n *represents the ratio between the number of samples and the number of genes. In (a) the comparison of the Kullback-Leibler divergences of IRIS (red asterisk line) and EM-MAP (blue crossed line) obtained for different values of the ratio *m*/*n *(*x*-axis). In (b) the percentage of correct (blue bars), undefined (green bars) and incorrect (red bars) evaluations of IRIS for different *m*/*n *ratio values.

Because MYC-subnetwork has an acyclic structure (differently to *S. cerevisiae *cell cycle) we can use EM-MAP algorithm to infer the PTs for this network. In order to evaluated the method in terms of scalability, Figure 9(a) reports the Kullback-Leibler divergences obtained by IRIS (red asterisk line) and EM-MAP (blue crossed line) for different values of *m*/*n *(*m *is the number of samples and *n *is the number of genes). As can be seen from the Figure, as the ratio *m*/*n *increases the accuracy gets better. This is also evident from Figure 9(b) where the percentage of correct (blue bars), undefined (green bars) and incorrect (red bars) evaluations of IRIS algorithm are reported.

Table 6 shows the results obtained by IRIS on the complete data set composed by 100 samples. Among the genes in MYC-DB, we obtained 93% of correct evaluations. Genes with no specified regulation in MYC-DB such as *EEF1E1, TRAP1 *and *PAICS *resulted up-regulated in IRIS. In particular for *PAICS *this up-regulation was confirmed in [39]. In addition there are 31 genes in the Basso *et al. *network with no MYC-DB entries, IRIS identifies 24 regulatory relationships that deserve further biological investigation.

**Table 6:** IRIS results for the *MYC *subnetwork including 55 genes directly connected to *MYC*.

Gene	IRIS Inferred Regulation	MYC-DB Regulation
*HSPC111*	Upregulation	Upregulation

*PPAT*	Upregulation	Upregulation

*POLD2*	Upregulation	Upregulation

*NOL5A*	Upregulation	Upregulation

*ZRP1*	Downregulation	Upregulation

*NME1*	Undefined	Upregulation

*EBNA1BP2*	Upregulation	Upregulation

*APEX1*	Upregulation	Upregulation

*NDUFB5*	Undefined	Not Specified

*PSPH*	Upregulation	Upregulation

*EEF1E1*	Upregulation	Not Specified

*CTPS*	Upregulation	Upregulation

*C1QBP*	Upregulation	Upregulation

*SRM*	Undefined	Upregulation

*CCT3*	Upregulation	Upregulation

*NOLC1*	Upregulation	Upregulation

*JTV1*	Upregulation	Upregulation

*TRAP1*	Upregulation	Not Specified

*BOP1*	Undefined	Upregulation

*IARS*	Upregulation	Upregulation

*EIF3S9*	Upregulation	Upregulation

*PAICS*	Upregulation	Not Specified

*RRS1*	Upregulation	Upregulation

*RCL*	Undefined	Upregulation

*POLR1C*	Upregulation	Not Present

*DKFZP564M182*	Downregulation	Not Present

*HPRT1*	Upregulation	Not Present

*C4orf9*	Upregulation	Not Present

*MRPL9*	Upregulation	Not Present

*LOC283537*	Downregulation	Not Present

*STAT3*	Downregulation	Not Present

*TUFM*	Undefined	Not Present

*SUPV3L1*	Upregulation	Not Present

*MRPL3*	Upregulation	Not Present

*LIMK2*	Upregulation	Not Present

*ATP6VOD1*	Downregulation	Not Present

*MX1*	Upregulation	Not Present

*TOMM40*	Upregulation	Not Present

*CYC1*	Upregulation	Not Present

*NOLA2*	Upregulation	Not Present

*MRPL12*	Upregulation	Not Present

*TIP-1*	Undefined	Not Present

*BYSL*	Upregulation	Not Present

*PFAS*	Upregulation	Not Present

*ZT86*	Undefined	Not Present

*TRA@*	Undefined	Not Present

*PRMT3*	Upregulation	Not Present

*MGC27165*	Undefined	Not Present

*ATIC*	Upregulation	Not Present

*HSD17B8*	Upregulation	Not Present

*SSRP1*	Undefined	Not Present

*TEGT*	Downregulation	Not Present

*TCP1*	Upregulation	Not Present

*Cdna_flj30991*	Undefined	Not Present

*IDH3A*	Upregulation	Not Present

Note: *Not Specified *indicates a gene for which an entry exists in MYC-DB but for which no information on regulation is available; *Not Present *indicates a gene for which no entry in MYC-DB exists; *Undefined *indicates a situation for which IRIS cannot distinguish between up- and down-regulation.

## Conclusions

This paper described a method to infer regulatory relations in gene networks from expression data. The basic features of IRIS are a simple discretisation method to translate real-valued measurements into two discrete states (active and inactive) and a regulatory inference rule. To compare the proposed approach with other methods, we reported results for synthetic networks. The main conclusion is that the proposed method yields similar or slightly better results than other well-known approaches, but requires much less computational resources.

We also tested IRIS on two real data sets to infer interaction rules for the yeast mitotic cell cycle and the human *MYC *sub-network. IRIS exhibited good accuracy for these networks compared to literature-derived rules. IRIS relies on knowledge of the network topology, which can be extracted from on-line databases (e.g. KEGG) or can be obtained from network reverse engineering algorithms. In other words regulatory network parameter estimation and model selection are treated and performed as two different tasks. This approach could be useful in studying gene regulatory networks with hundreds of genes as a set of smaller sub-networks, as reported for *MYC *expression profiles.

IRIS is useful for extracting the main rules within a gene network with a well-defined topology. This information can then be used in subsequent analysis steps, such as probabilistic inference or as a preliminary step for building models of complex biological systems [14].

## Availability and Requirements

• **Project name**: Inference of Regulatory Interaction Schema (IRIS)

• **Project home page**: http://bioinformatics.biogem.it:8081/BioPlone/downloadfolder/iris-download-page

• **Operating system(s)**: Platform independent

• **Programming language**: MATLAB

• **Other requirements**: *Graphviz *is required if the user wants to create a file containing the gene regulatory network topology

• **License**: GNU GPL

• **Any restrictions to use by non-academics**: None

## Authors' contributions

SM performed all the experiments and wrote the code, PZ helped in devising the experiments and analysing the results, and MC proposed the biological problem and devised the method. All authors contributed to the design of the whole work and to the writing of the manuscript. All authors read and approved the final manuscript.

## Supplementary Material

Additional file 1 This is a pdf file that lists the true descriptions of the regulatory functions of all the gene regulatory networks used in this paper. Click here for file
