# Supplementary material for: IRIS: a method for reverse engineering of regulatory relations in gene networks
Source: BMC Bioinformatics. 2009 Dec 23;10:444. doi: 10.1186/1471-2105-10-444 (PMC2813854; doi:10.1186/1471-2105-10-444)
Supplement: Additional file 1 — This is a pdf file that lists the true descriptions of the regulatory functions of all the gene regulatory networks used in this paper. [file 1471-2105-10-444-S1.PDF]

Regulation Function True Descriptions

Sandro Morganella Pietro Zoppoli Michele Ceccarelli

October 14, 2009

Abstract

In this addictional file you found the true descriptions of all regulation functions used within the reference paper.

0.1 *Escherichia Coli* Synthetic Network

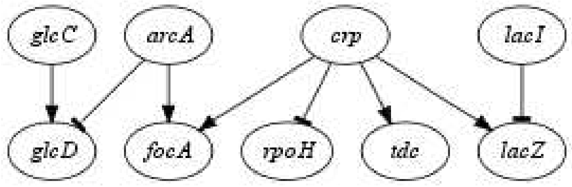

Figure 1: *E. coli* synthetic network.

(a)

glcC	*arcA*	*glcD*
0	0	0
0	1	0
1	0	1
1	1	0

(b)

arcA	*crp*	*focA*
0	0	0
0	1	1
1	0	1
1	1	1

(c)

crp	*lacI*	*lacZ*
0	0	0
0	1	0
1	0	1
1	1	1

(d)

crp	*rpoH*
0	1
1	0

(e)

crp	*tdc*
0	0
1	1

Table 1: True description of regulatory relations in *E. coli* synthetic network.

0.2 Yeast *Saccharomyces Cerevisiae* Synthetic Network

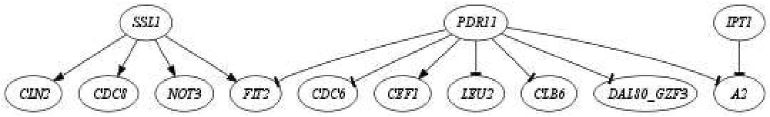

Figure 2: *S. cerevisiae* synthetic network.

(a)

PDR11	*SSL1*	*FIT2*
0	0	0
0	1	1
1	0	0
1	1	0

(b)

PDR11	*IPT1*	*A2*
0	0	1
0	1	0
1	0	0
1	1	0

(c)

SSL1	*CLN2*
0	0
1	1

(d)

SSL1	*CDC8*
0	0
1	1

(e)

SSL1	*NOT3*
0	0
1	1

(f)

PDR11	*DAL80_GZF3*
0	1
1	0

(g)

PDR11	*CDC6*
0	1
1	0

(h)

PDR11	*CEF1*
0	0
1	1

(i)

PDR11	*LEU2*
0	1
1	0

(j)

PDR11	*CLB6*
0	1
1	0

Table 2: True description of regulatory relations in *S. cerevisiae* synthetic network.

0.3 Cell Cycle network of *Saccharomyces Cerevisiae*

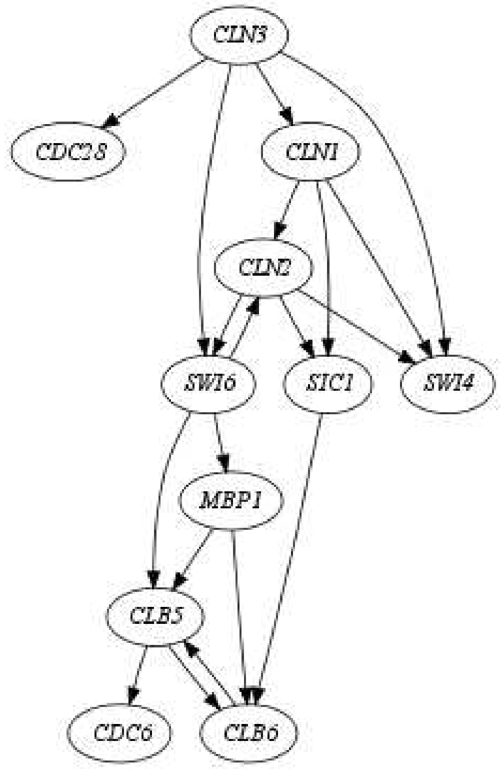

Figure 3: Cell Cycle network of *Saccharomyces Cerevisiae*.

(a)

SWI6	*MBP1*	*CLB6*	*CLB5*
0	0	0	0
0	0	1	1
0	1	0	0
0	1	1	1
1	0	0	0
1	0	1	0
1	1	0	0
1	1	1	1

(b)

CLN3	*CLN1*	*CLN2*	*SWI4*
0	0	0	0
0	0	1	1
0	1	0	0
0	1	1	1
1	0	0	1
1	0	1	1
1	1	0	1
1	1	1	1

(c)

MBP1	*SIC1*	*CLB5*	*CLB6*
0	0	0	0
0	0	1	0
0	1	0	0
0	1	1	0
1	0	0	0
1	0	1	1
1	1	0	0
1	1	1	1

(d)

CLN3	*CLN2*	*SWI6*
0	0	0
0	1	1
1	0	1
1	1	0

(e)

SWI6	*CLN1*	*CLN2*
0	0	0
0	1	1
1	0	0
1	1	1

(f)

CLN1	*CLN2*	*SIC1*
0	0	1
0	1	1
1	0	1
1	1	0

(g)

CLN3	*CLN1*
0	0
1	1

(h)

CLN3	*CDC28*
0	0
1	1

(i)

SWI6	*MBP1*
0	0
1	1

(j)

CLB5	*CLB6*
0	0
1	1

Table 3: True description of regulatory relations in *S. cerevisiae* cell-cycle.
